# Cutaneous innervation and trigeminal pathway function in a patient with facial pain associated with Parry-Romberg syndrome

**DOI:** 10.1007/s10194-012-0459-0

**Published:** 2012-05-24

**Authors:** M. Falla, A. Biasiotta, G. Fabbrini, G. Cruccu, A. Truini

**Affiliations:** 1 Department of Neurology and Psychiatry, Sapienza University, Viale Università 30, 00185 Rome, Italy; 2 Don Gnocchi Foundation, Milan, Italy

**Keywords:** Parry-Romberg syndrome, Hemifacial atrophy, Trigeminal nerve

## Abstract

Parry-Romberg syndrome (PRS) is a rare condition manifesting with progressive hemifacial atrophy. Although reported PRS clinical disturbances include facial pain and recent studies raised the possibility that PRS-related pain is a neuropathic pain condition due to the trigeminal nerve damage, no studies have directly investigated cutaneous innervation and trigeminal pathway function in patients with this rare condition. In a 50-year-old woman presenting with a 10-year history of slowly progressive hemifacial atrophy and facial pain, we investigated large myelinated fibres with masticatory muscle electromyography and trigeminal reflexes, and tested small myelinated and unmyelinated fibres with laser-evoked potentials. We also investigated cutaneous innervation by measuring the intraepidermal nerve fibre (IENF) density after skin biopsy of the supraorbital regions. We found that neurophysiological data and IENF density came within normal ranges, with no differences between normal and affected side. Our study showing that the standard reference techniques for assessing cutaneous innervation and trigeminal pathway function disclosed no abnormalities in this patient with PRS suggest that this rare and disabling condition is not associated with trigeminal system damage. These findings indicate that in this patient PRS-related pain is not a neuropathic pain condition, rather it probably arises from the musculoskeletal abnormalities.

## Introduction

Parry-Romberg syndrome (PRS) is a rare condition manifesting with progressive hemifacial atrophy involving skin, soft tissue, and bone. Reported PRS clinical disturbances include facial pain [1, 2]. The documented inflammatory changes in the brain parenchyma and vessel walls, occasional coexisting autoimmune disorders and clinical improvement following immunosuppression suggest that PRS could be an immune-mediated disease [3]. Despite recent studies suggesting that PRS-related pain is a neuropathic pain condition due to trigeminal nerve damage, no studies have directly investigated cutaneous innervation and trigeminal pathway function in patients with this rare condition. Having this information might help to understand the underlying causes of PRS, thus opening the way to a therapeutic approach.

## Case presentation

A 50-year-old woman presented with a 10-year history of slowly progressive hemifacial atrophy associated with facial pain. Her medical history was unremarkable except for high blood pressure. Clinical examination showed facial asymmetry with marked hypoplasia involving the right face, and right enophthalmos. The patient complained of a dull, aching pain affecting the right face. Pain was continuous, deeply and poorly localised, mainly distributed to the supra- and periorbital regions, temple, ear, and zygomatic area. Pain was mild in intensity, with no significant variations during the day. The remaining clinical and neurological examinations were normal, including a detailed sensory examination using bedside tools. The soft brush and the pinprick stimulation of the skin did not evoke pains (namely dynamic mechanical allodynia and hyperalgesia). We assessed large myelinated fibres with masticatory muscle electromyography and trigeminal reflexes (blink reflex and masseter inhibitory reflex) [4], and tested small myelinated and unmyelinated fibres (Aδ- and C-fibres) with laser-evoked potentials [5]. We also investigated cutaneous innervation by measuring the intraepidermal nerve fibre (IENF) density after skin biopsy of the supraorbital regions. The methods used adhered to European Guidelines [6], and published recommendations [7, 8]. The blink reflex was evoked by electrical stimulation of the supraorbital nerve. EMG signals were recorded from the orbicularis oculi through surface electrodes. The masseter inhibitory reflex was evoked by electrical stimulation of the infraorbital and mental nerves, while the subjects were instructed to clench the teeth at maximum strength with the aid of auditory feedback. EMG signals were recorded from the masseter muscle through surface electrodes. To study laser-evoked potentials we used a previously reported technique [9]. The main Aδ- and C-LEP complex, N2–P2, was recorded through disc electrodes from the vertex (Cz) referenced to the nose. We measured peak latency and amplitude (peak-to-peak) of the main N2–P2 vertex complex.

For sampling skin innervated by the trigeminal nerve, after local anaesthesia 2-mm punch biopsies were taken above the eyebrow. Samples were processed for bright-field immunohistochemistry, using antibodies against protein gene product (PGP) 9.5, as a marker for intraepidermal nerve fibres. Neurophysiological and IENF data were compared with normative ranges established in our laboratory.

**Fig. 1:**
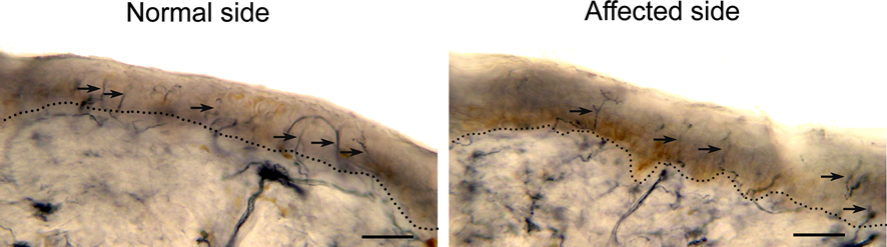
Skin biopsy from the normal and affected supraorbital region. Intraepidermal nerve fibres (IENF) immunostained with the panaxonal marker anti-protein gene product 9.5 in a 50-μm skin section. *Arrows* indicate normal IENF crossing the dermo-epidermal junction. *Scale bar* 50 μm. The IENF density was 13.1/mm at the normal supraorbital region and 15.1/mm at the affected supraorbital region

**Fig. 2:**
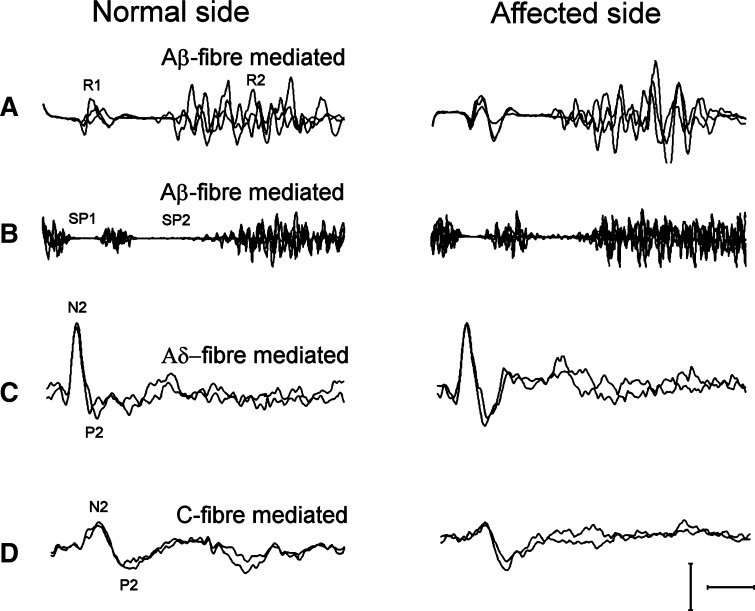
Neurophysiological testing after stimulation of the normal and affected side. Blink reflex (**a**) after supraorbital nerve stimulation and masseter inhibitory reflex (**b**) after infraorbital nerve stimulation. Three trials superimposed. Calibration 10 ms/200 μV for **a**, 20 ms/200 μV for **b**. Aδ- (**c**) and C-fibres (**d**) related laser-evoked potentials after supraorbital stimulation. Two averages of ten trials each superimposed. Calibration 200 ms/20 μV. The latency and the amplitude of short (*R1* and *SP1*) and long latency (*R2* and *SP2*) trigeminal reflex responses, and vertex components (*N2* and *P2*) of laser-evoked potentials were within normal ranges, with no side asymmetry

Laboratory testing showed no abnormalities. Investigation for rheumatic disease, including rheumatoid factor, anti-dsDNA antibody, extractable nuclear antigen screening were normal. Computerised tomographic (CT) and magnetic resonance imaging (MRI) scans showed severe hemiatrophy involving facial subcutaneous tissues and facial bones, but no brain lesions. Neurophysiological data and IENF density came within normal ranges, with no differences between normal and affected side (Figs. 1, 2).

## Discussion

Our study showing that the standard reference techniques for assessing cutaneous innervation and trigeminal pathway function disclosed no abnormalities in a patient with PRS and facial pain suggest that in our patient this rare and disabling condition is not associated with trigeminal system damage. This implies that in this patient affected by PRS, the facial pain is not a neuropathic pain condition. The recent criteria for diagnosing neuropathic pain assume that a definite diagnosis of neuropathic pain requires confirmatory evidence from neurologic examination and laboratory investigation showing the damage of the somatosensory afferent pathway [10].

Previous studies raised the possibility that PRS-related pain is a neuropathic pain condition [1, 2]. In these studies the diagnosis of neuropathic pain relied on clinical examination and quantitative sensory testing (QST) abnormalities showing hyperalgesia and allodynia [2]. Although QST abnormalities are also found in non-neurological diseases and they cannot be taken as a conclusive demonstration of neuropathic pain [11, 12], and these studies did not perform an objective assessment of trigeminal afferent pathway, we cannot exclude that our patient presented with a different phenotype. Indeed the patient reported by Viana and colleagues also suffered from a severe, shooting pain resembling trigeminal neuropathic pain.

According to our data we hypothesise that in our patient PRS-related pain is a nociceptive pain condition that might arise from the musculoskeletal abnormalities. These abnormalities might be directly due to the immune-mediated damage of soft tissues and bone or a consequence of the musculoskeletal impairment affecting temporomandibular joint and masticatory muscles.

Further studies assessing trigeminal pathway function are needed to verify whether PRS-related pain is invariably a nociceptive pain condition or different type of pain (namely neuropathic pain) may occur.
